# Case report: Treatment of congenital lobar emphysema with lung lobectomy in a puppy

**DOI:** 10.3389/fvets.2023.1083376

**Published:** 2023-06-27

**Authors:** Lauren M. Edwards, Cassie N. Lux, Matthew Everett, Silke Hecht

**Affiliations:** Department of Small Animal Clinical Sciences, University of Tennessee College of Veterinary Medicine, Knoxville, TN, United States

**Keywords:** canine, lung lobectomy, lobar emphysema, congenital, computed tomography

## Abstract

An 11-week-old, sexually intact female Catahoula Leopard dog was evaluated for a multiple-week history of exercise intolerance and intermittent periods of respiratory distress. Thoracic radiographs revealed a markedly hyperinflated right lung field, with compression of the surrounding lung lobes. Thoracic computed tomography further localized the hyperinflation to the right middle lung lobe, with suspicion of congenital lobar emphysema. A right intercostal thoracotomy with right middle lung lobectomy was performed successfully. Histopathology results confirmed bronchial cartilage hypoplasia with marked emphysema and pleural fibrosis. The puppy recovered from surgery uneventfully and was discharged from the hospital without any postoperative complications. At 18 months postoperatively, the dog was clinically normal with no return of respiratory distress. This case report describes successful surgical treatment of a large breed puppy with the uncommonly reported condition of congenital lobar emphysema.

## Introduction

Congenital lobar emphysema (CLE) is a rare lower respiratory tract disease that most commonly presents in young dogs and cats, with age ranges in the literature from 6 weeks to 24 months of age (1–8). The literature predominately reports CLE in small or toy breed dogs, with rare reports in large breed dogs (9). Clinical signs reported in the literature include respiratory signs such as exercise intolerance, coughing, tachypnea or dyspnea, and cyanosis (1, 3–6, 8–13). Additionally, subcutaneous emphysema, pneumothorax, and pneumomediastinum have been noted on imaging and physical examination (3, 4, 7, 8, 13, 14). Congenital lobar emphysema is characterized by alveolar air accumulation resulting in hyperinflation of the affected lung lobes, most commonly due to bronchial cartilage hypoplasia, dysplasia, or aplasia leading to bronchial collapse (1–6, 8, 10, 11, 13–18). In the human literature, there are 3 recognized etiologies of lobar emphysema: bronchial cartilage dysplasia, which may range from hypoplastic and flaccid cartilage to a complete absence of tissue; external bronchial compression; and idiopathic (19, 20). Idiopathic etiologies have also been reported in veterinary patients, in which no bronchial abnormalities were identified on histopathology (7, 9, 10, 12). While many cases reported in the veterinary literature have bronchial abnormalities, ~80% of people diagnosed with CLE have an undetermined etiology, and only 20% have associated bronchial abnormalities (19). Surgical intervention with lung lobectomy of the affected lobes has been reported to result in successful treatment and recovery; however, older literature predominantly reported death or euthanasia in dogs affected with CLE (10–14, 16, 21). The case report described here details diagnosis and treatment of a large-breed puppy with CLE.

## Case presentation

**Figure 1:**
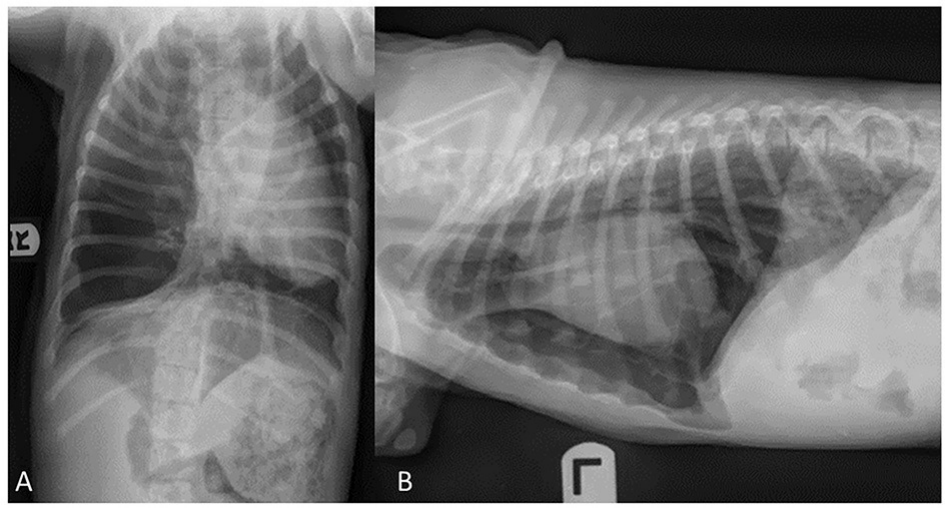
Ventrodorsal **(A)** and lateral **(B)** radiographic projections of an 11-week old, sexually intact, female Catahoula Leopard dog with congenital lobar emphysema. Noted in image **(A)**, is a left mediastinal shift, and images **(A, B)** depict a hyperlucent right lung lobe and displacement of the cardiac silhouette.

Informed consent was obtained from the owner for publication of this case report. An 11-week-old, sexually intact, female Catahoula Leopard dog with a weight of 6 kg was evaluated for a multiple-week history of exercise intolerance and intermittent periods of respiratory distress. At 8 weeks of age, the primary veterinarian performed thoracic radiographs to investigate for causes of exercise intolerance and respiratory distress (Figures 1A, B). The thoracic radiographs revealed a markedly hyperinflated and hyperlucent right lung lobe with compression of the surrounding lobes, displacement of the cardiac silhouette, and a leftward mediastinal shift. The dog was subsequently referred to a specialty hospital for further diagnostics and care with a differential diagnosis including congenital lobar emphysema based on the above radiographic changes.

On presentation to the soft tissue surgery service at the referral hospital, physical examination revealed absent right lung sounds and increased, harsh left lung sounds on thoracic auscultation. The dog's respiratory effort was moderately increased on presentation. Her temperature (100.4° F, 38°C), pulse (150 beats per min), and the remainder of her physical examination were within normal limits. Bloodwork abnormalities were consistent with the young age of the patient, including a mild anemia (hematocrit 35.1%; reference range 40.5–59.9%), lymphocytosis (lymphocytes 5.18 x 10^3^/μL; reference range 1.10–3.96 x 10^3^/μL), mild panhypoproteinemia (albumin 2.9 g/dL; reference range 3.2–4.3 g/dL and globulins 1.5 g/dL; reference range 1.9–3.1 g/dL), hyperphosphatemia (7.8 mg/dL; reference range, 2.5–5.9), hyperkalemia (4.9 mmol/L; reference range, 2.-4.7 mmol/L) and a mild elevation in ALP (295 U/L; reference range, 13–240 U/L). To further characterize the pulmonary pathology, a thoracic computed tomography (CT) scan was performed under general anesthesia with the intention to proceed directly to surgery pending the results of the CT scan. The patient was sedated with an intramuscular injection of butorphanol (0.4 mg/kg) and alfaxalone (2 mg/kg), after which anesthesia was induced with an intravenous injection of ketamine (5 mg/kg) and midazolam (0.25 mg/kg). General anesthesia was maintained with vaporized sevoflurane and continuous rate infusions of ketamine (10–20 mg/kg/h) and fentanyl (5–10 μg/kg/h).

A transverse multislice submillimeter helical dataset was obtained from the thoracic inlet to the cranial abdomen with a 40-slice helical CT scanner (Philips Brilliance-40, Philips International B.V., Amsterdam, Netherlands). To decrease risk of pulmonary rupture and pneumothorax, a breath hold was not utilized when acquiring the CT images. A pre- and post-contrast study was completed using iodinated contrast material (Optiray, 0.45 mL/kg of 350 mg I/mL IV).

Images revealed a severely distended right middle lung lobe with hypoattenuating parenchyma relative to the remaining lung lobes. Furthermore, the pulmonary vessels throughout the right middle lung lobe were decreased in size and the primary bronchus was mildly dilated, with abrupt narrowing in the distal bronchus.

**Figure 2:**
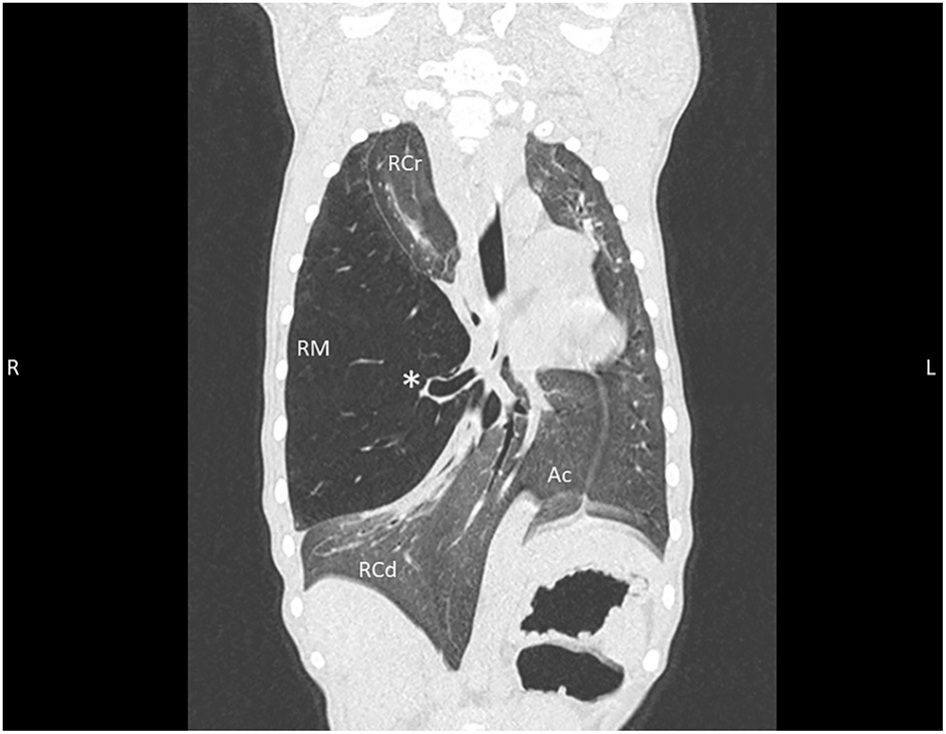
Thoracic CT dorsal reconstruction images from an 11-week old, sexually intact, female Catahoula Leopard dog with congenital lobar emphysema. Note the hyperinflated right middle lung lobe (RM) with an abrupt narrowing of the main stem bronchus (asterisk); leftward deviation of the heart, mediastinal structures, and accessory lung lobe (Ac); and diffuse increased attenuation/ground glass pattern within remaining right cranial (RCr) and right caudal (RCd) lung lobes as well as left lung.

Distension of the right middle lung lobe resulted in a marked leftward displacement of the heart and other mediastinal structures as well as the right cranial, right caudal and accessory lung lobes (Figure 2). Consequential compression of the left caudal and left cranial lung lobes also occurred. Multifocal regions of increased pulmonary attenuation and ground glass patterns throughout the displaced and compressed lung lobes were noted and attributed to atelectasis. A diagnosis of CLE was suspected based on hyperinflation of the right middle lung lobe and abrupt narrowing of the bronchus within the right middle lung most likely related to bronchial hypoplasia and less likely bronchial compression. The remaining changes of the thoracic cavity were deemed likely to be secondary to compression and displacement from the right middle lung lobe.

With careful consideration for the age of the patient and prognosis without treatment, it was elected to continue with surgical intervention. The dog proceeded to surgery immediately following the CT scan under the same anesthetic event. A fentanyl loading dose of 5 μg/kg was administered intravenously, and Normosol-R intravenous fluids were administered at a rate of 3 ml/kg/h. In addition to the vaporized sevoflurane, continuous rate infusions of ketamine (10–20 mg/kg/h) and fentanyl (5–10 μg/kg/h) were utilized to maintain a surgical plane of anesthesia.

Following aseptic preparation of the right lateral thoracic wall, the patient was positioned for surgery in left lateral recumbency. Mechanical ventilation was started prior to incision of the thoracic wall. A right lateral intercostal thoracotomy was performed with a 12 cm incision made in a dorsoventral direction along the 5^th^ intercostal space. A standard intercostal thoracotomy approach with sharp and blunt dissection of the subcutaneous tissues and thoracic wall musculature was performed, and finochietto retractors were used for retraction of the ribs to aid in visualization.

**Figure 3:**
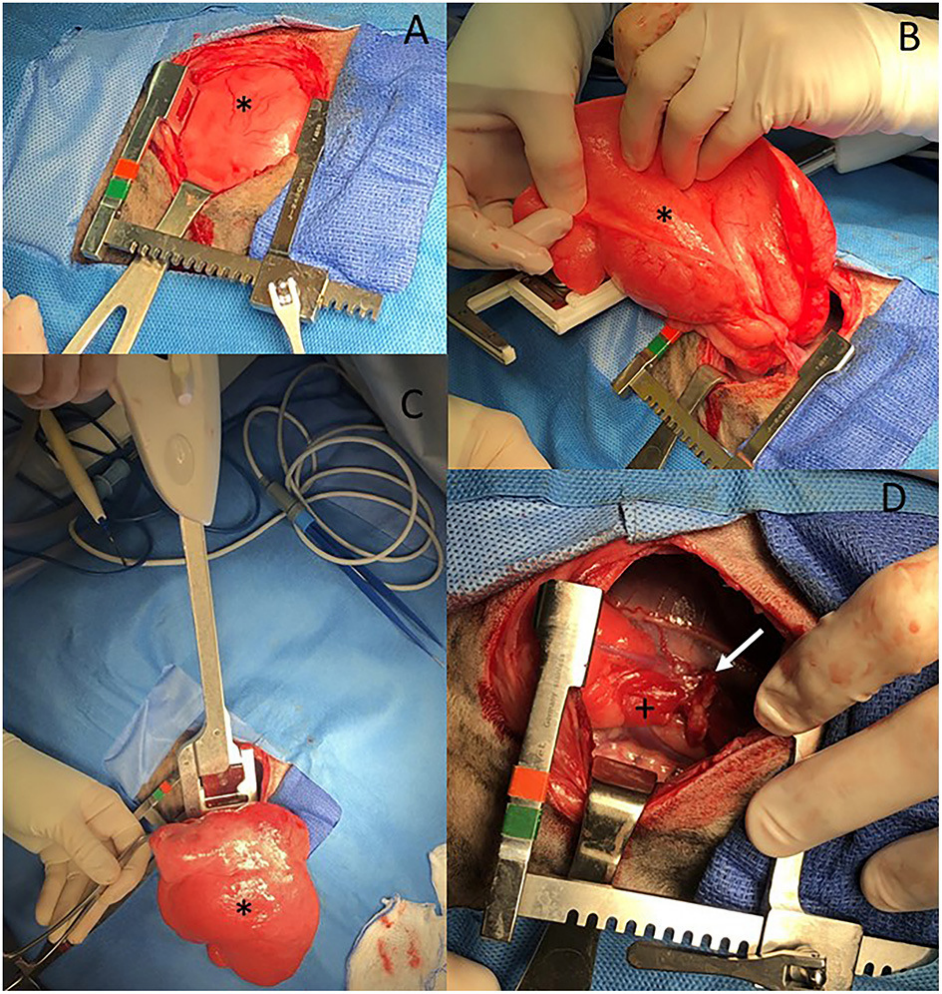
**(A–D)** Intraoperative images from an 11-week old, sexually intact, female Catahoula Leopard dog with CLE. The head is to the right and dorsal is on the bottom of all images. The right middle lung lobe (asterisk in all images) is occupying a majority of the right thoracic cavity **(A)** and is hyperinflated **(A, B)**. A lung lobectomy was performed with a DST Series TA Stapler using a 30 mm V3 vascular stapler **(C)**. The right middle lung hilus can be seen with the staple line (white arrow) following lobectomy, and the right caudal lung lobe has an area of atelectasis (+) **(D)**.

The markedly inflated right middle lung lobe was manipulated to access the hilus, resulting in a majority of the lobe being externalized from the thoracic cavity (Figure 3). A DST Series^TM^ TA^TM^ Stapler (Medtronic, Minneapolis, MN) with a 30 mm V3 Vascular (Medtronic, Minneapolis, MN) cartridge was used to seal the bronchus, artery and vein at the hilus of the right middle lung lobe. A scalpel was then used to excise the lung lobe distal to the staples. An exploratory exam of the right hemithorax was performed and was within normal limits except for notation of an atelectatic right caudal lung lobe.

A local block of bupivacaine (6 mg; 1 mg/kg) and lidocaine (6 mg; 1 mg/kg) was placed into the intercostal muscles cranial to, at the level of, and caudal to the incision. A red rubber catheter connected to a 3-way stopcock and 20 mL syringe was passed through the incision prior to closure for evacuation of air from the thorax. The ribs were apposed using simple interrupted sutures of 3-0 PDS, and the musculature of the thoracic wall was apposed with 3-0 Monocryl in a simple continuous pattern. Prior to closure of the skin, the anesthetist was consulted as to whether substantial resistance was noted during hand-ventilation. Air was suctioned from the thoracic cavity until anesthesia noted minimal resistance to ventilation. Negative pressure was not re-established within the thoracic cavity due to concern of re-expansion injury to the remaining lung lobes. The thoracic catheter used for air evacuation was removed prior to closure of the skin, and the skin was apposed in an intradermal pattern using 4-0 Monocryl.

**Figure 4:**
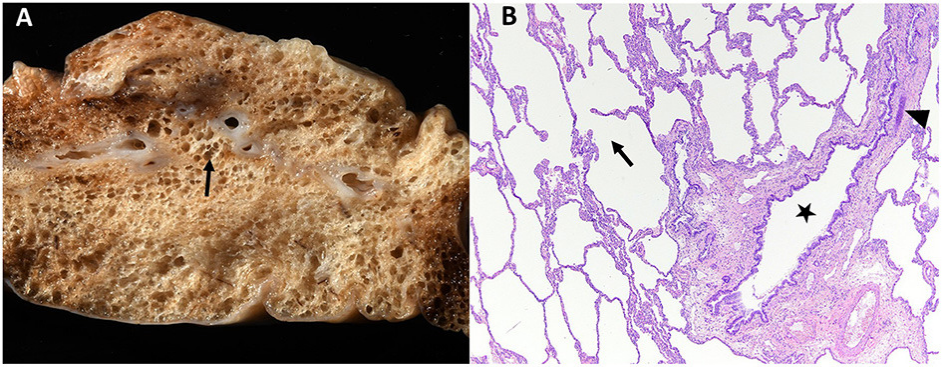
Gross **(A)** and histopathologic **(B)** images of the right middle lung lobe affected by CLE are depicted. Ruptured alveoli are present in the lung lobe: grossly this is visible as air pockets within the lung tissue (surrounding the arrow, **A**) and microscopically as discontinuity of the alveolus wall (arrow within alveolus, **B**). In image **(B)**, bronchial cartilage is present as a small discontinuous basophilic band (arrow head), which should fully surround a normal bronchus. This represents bronchial cartilage hypoplasia and is the cause of the bronchus in image **(B)** (star) being partially collapsed instead of rounded.

**Figure 5:**
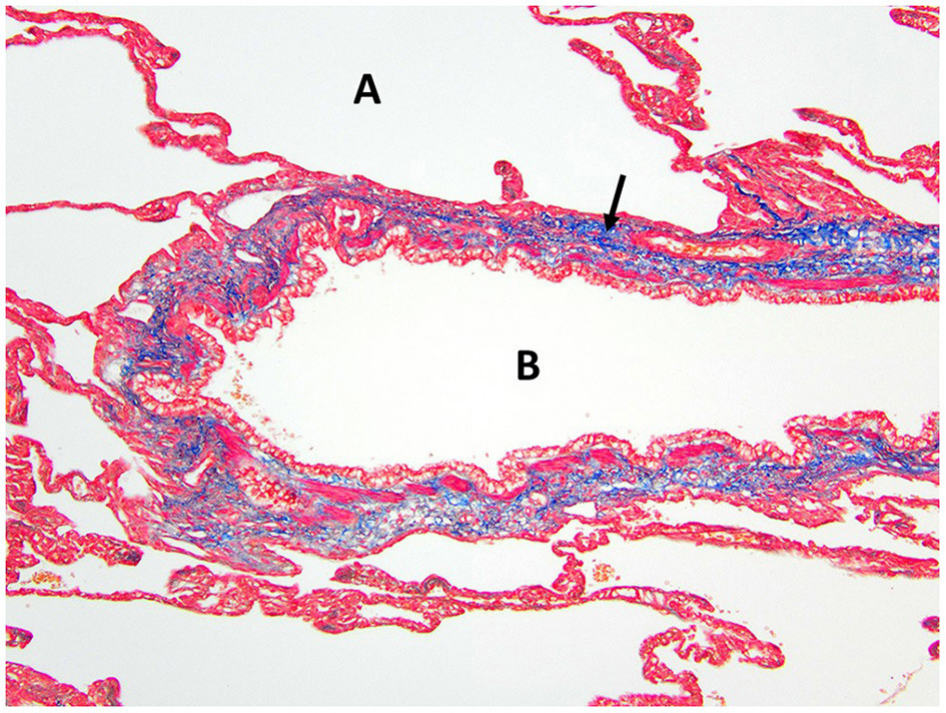
Masson's trichrome staining of the right middle lung lobe highlights the abundant collagen/connective tissues (arrow) surrounding the bronchus B with no cartilage present. A, alveolus.

**Figure 6:**
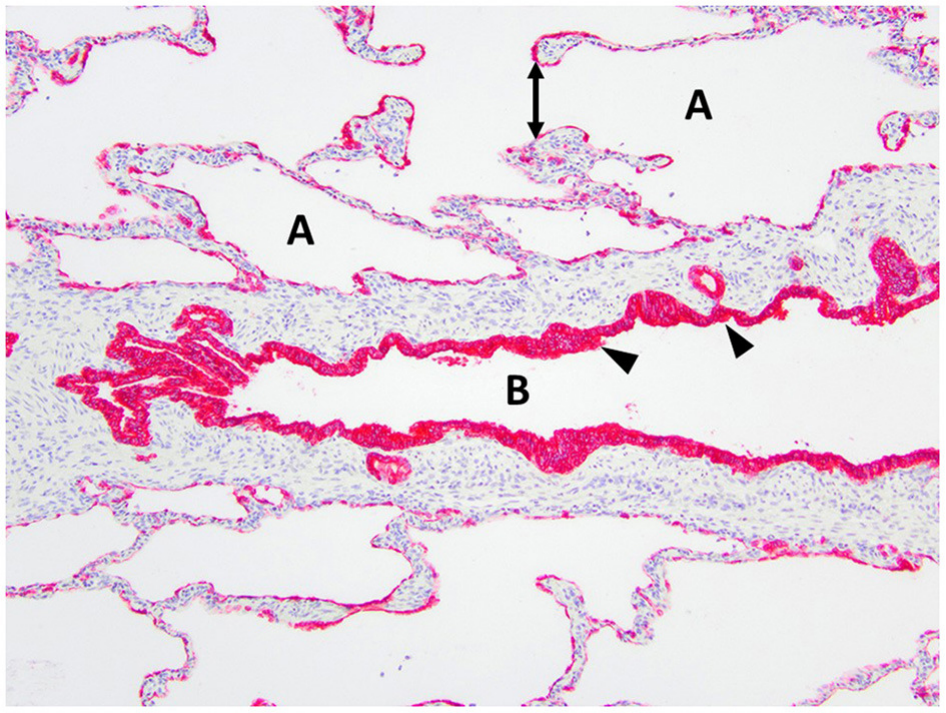
Immunohistochemistry AE1/AE3 cytokeratin of the right middle lung lobe highlights the bronchial epithelium with multilayered columnar to cuboidal epithelium and invaginations (arrowheads). The bronchus is identified as B. The alveoli are identified as A and contain ruptured septa with clubbed ends (double arrow).

**Figure 7:**
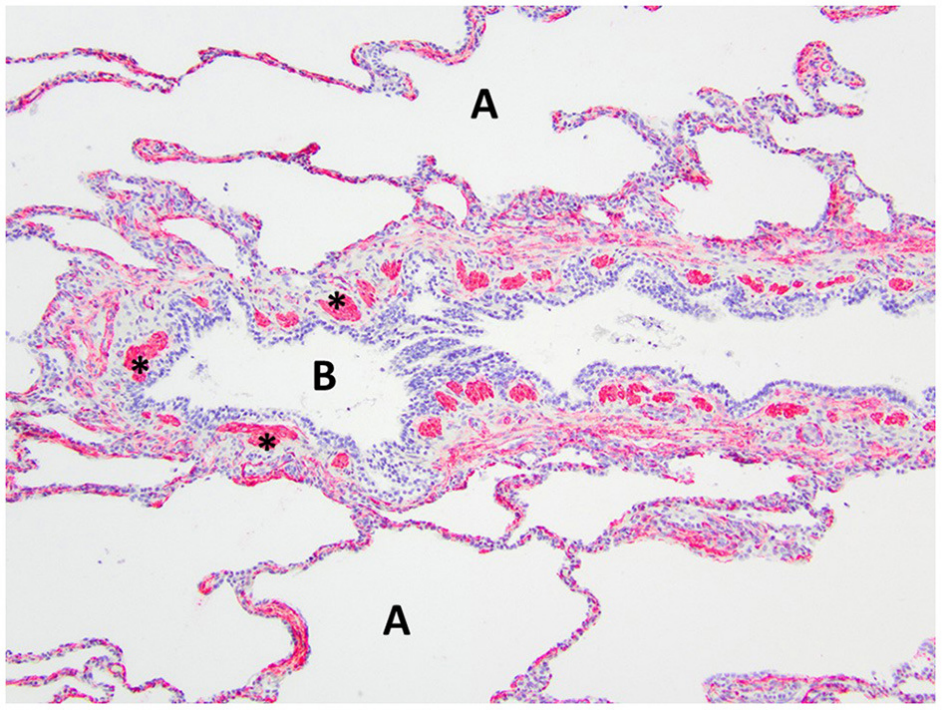
Immunohistochemistry α-SMA for smooth muscle of the right middle lung lobe highlights the abnormal bronchial smooth muscle architecture as the discontinuous smooth muscle bundles (asterisks) surrounding the bronchus B. A, alveoli.

Histopathology was performed including hematoxylin and eosin (H&E) stain, Masson's trichrome stain to identify collagen, and immunohistochemistry antibodies for AE1/AE3 cytokeratin to identify epithelial cells and α-smooth muscle actin (α-SMA) to evaluate bronchial smooth muscle of the resected right middle lung lobe (Figures 4–7). Results revealed bronchial cartilage hypoplasia characterized by variable quality cartilage to the absence of cartilage, dilated and coalescing alveoli consistent with marked emphysema, abnormal bronchial smooth muscle architecture, and expanded connective tissues and collagen with pleural fibrosis (Figures 4–7). The microscopic findings were consistent with the suspected clinical diagnosis of congenital lobar emphysema.

The dog recovered uneventfully from anesthesia and was maintained on a fentanyl constant rate infusion (2–5 μg/kg/h IV, titrated to effect), gabapentin (10 mg/kg PO every 8 h), and carprofen (2 mg/kg PO every 12 h). Oxygen supplementation at an FiO_2_ of 30–40% was provided for 8 h via an oxygen cage, and the dog had a normal respiratory rate and effort the following day out of oxygen supplementation. The dog was discharged 24 h postoperatively with no apparent complications. The owners reported the dog continued to recover well from surgery. As of 18 months postoperatively, the dog was doing clinically well, with no dyspnea or exercise intolerance per owner communication.

## Discussion

This case report details the clinical treatment of a young dog diagnosed with CLE and undergoing successful surgical management of this condition. The diagnostic imaging, surgical, and histopathologic findings were all considered characteristic of CLE. Congenital lobar emphysema is a rare lower respiratory tract disease that is characterized by alveolar air accumulation with resulting hyperinflation of the affected lung lobes. Air accumulation is believed to be due to dynamic airway collapse, in which entry of air on inspiration occurs normally, however, abnormal bronchial collapse during expiration does not allow air to escape (1, 2, 9, 13, 19). It is this overdistension due to hyperinflation that causes the lung to be generally non-functional. Abnormal bronchial collapse and obstruction on expiration has been reported to be associated with bronchial cartilage hypoplasia, dysplasia, and aplasia; external bronchial compression; and idiopathic etiologies (1–7, 10–12, 14, 17, 18).

Histopathology of the lung lobe removed from the dog reported here revealed bronchial cartilage hypoplasia with marked emphysema and pleural fibrosis. The microscopic findings of this lung lobe including the abundant collagen with no cartilage around the bronchus on the Masson's trichrome stain, the abnormal bronchial smooth muscle architecture on the α-SMA immunostaining, the cuboidal and invaginated epithelium lining the bronchus on the AE1/AE3 immunostaining, and the many areas of discontinuous ruptured alveolar septa were consistent with a diagnosis of CLE and previously reported (7, 8, 14). The pathologist commented that the deficient bronchial cartilage and bronchial atresia resulting in defects in the bronchial walls caused greater volumes of air to enter the affected lobe on inspiration than exited on expiration, resulting in air trapping and the clinical presentation of a hyperinflated lung lobe. The bronchial hypoplasia diagnosed here has been previously documented as associated with CLE in 10 reported cases in the literature (4–6, 14–17). Pleural fibrosis has been noted in other dogs with histopathologic analysis of the affected lung lobes, however the clinical significance of pleural fibrosis, as it pertains to CLE in dogs, is not described (7, 8, 14). Although some reported cases in dogs have determined CLE to be idiopathic in nature, it appears the dog in this report developed CLE due to the aforementioned bronchial abnormalities (7, 9, 10, 12).

The dog in this report was 11 weeks old at the time of surgery with clinical signs starting as early as 8 weeks old. Although CLE is often diagnosed in young animals, the dog in this report is one of the youngest dogs to have been clinically affected by CLE and successfully treated with surgical excision of the affected lung lobe. The 10 previous CLE patients with descriptions of successful surgical treatment consisted of nine dogs, ranging from 6 weeks to 10 years of age, and one 5-month-old kitten (1–9, 18). On evaluation of the previously reported cases, the median age at the time of successful treatment of CLE in the nine dogs was 20 weeks and was also 20 weeks in the only reported cat (1–9, 18). Importantly, this case report highlights a successful anesthetic and invasive surgical event for a pediatric puppy (< 12 weeks old), which comes with additional risks due to the immature age including reduced ability to respond to cardiovascular changes; immature renal, hepatic, and thermoregulatory functions; and reduced pulmonary reserve with high oxygen consumption (22, 23).

Of the 10 case reports with successful surgical treatment described previously, lobectomy has been performed on the following affected lung lobes in dogs and cats with CLE: 7/10 for the right middle lung lobe, 2/10 for the left caudal subsegment of the left cranial lung lobe, and 1/10 for the left caudal subsegment of the left cranial lung lobe and accessory lobe simultaneously (1–9, 18). A recent retrospective study of 14 dogs and 3 cats with lobar emphysema suspected a congenital etiology in 14/17 animals (17). In the same report, 8/17 animals underwent lung lobectomy with confirmed CLE diagnosis of the right middle lung lobe in all 8 cases, though one dog and one cat also had the right cranial and right caudal lobes affected, respectively (17). The dog in the case reported here, similar to other cases, was affected with CLE in the right middle lung lobe.

Although a variety of large-breed dogs with CLE have been reported (9, 11, 12, 17), descriptions of successful surgical intervention in the literature for large breed dogs are limited to a single case report of an Old English Sheepdog (9). Therefore, the description of successful surgical intervention for the dog in this report supplements the literature for surgical intervention in large-breed dogs. Although it is possible that other large-breed dogs were treated surgically, breed descriptions for dogs undergoing surgery were not available for one report (17). The majority of reports with successful surgical treatment are in small breed dogs (1, 3–8, 18).

Hyperinflation of the affected lung lobe on the thoracic radiographs resulted in pursuit of a CT scan to further define pulmonary pathology in the dog described here. Evidence of hyperinflation and hyperlucency of the affected lung lobes, mediastinal shift, pulmonary atelectasis, and elevation of the cardiac silhouette from the sternum were common findings in 15 previous cases that utilized radiography in their diagnosis of CLE (1–13, 16, 18). Of the 15 cases diagnosed by thoracic radiography and supporting clinical signs, only five went on to utilize CT for surgical planning (2, 3, 5, 6, 8). The CT scan in this dog revealed severely expanded pulmonary parenchyma with hypoattenuation compared to the surrounding lung lobes consistent with hyperinflation, which was also seen in all previously reported cases undergoing CT (2, 3, 5, 6, 8). The value of CT lies in the ability to diagnose other concurrent conditions in the pulmonary parenchyma and thoracic cavity such as pneumothorax, pneumomediastinum, bullae or blebs, bronchial abnormalities, and concurrent abnormalities in other lung lobes, which are all variably reported in the literature (2, 3, 5, 6, 8). It is important to note that multiple cases have been reported involving more than one lung lobe based on CT diagnosis (5, 17). Therefore, if a CT scan is not performed prior to surgical intervention, consideration should be made to approach the thoracic cavity via a median sternotomy instead of an intercostal thoracotomy if there is any concern that additional lung lobes on opposite sides of the thoracic cavity may be affected. This will allow for effective exploration of both hemithoraces. The additional findings in the dog of this case report due to use of the CT scan included abrupt narrowing of the bronchus in the affected lung lobe, compression with atelectasis of local lung lobes, and displaced thoracic structures.

It should be noted that not all patients are immature at the time of CLE diagnosis, and some patients may be asymptomatic for CLE (4, 8, 16–18). However, most patients do exhibit clinical signs related to CLE and at an immature age, similar to the dog of this report. Out of the total 18 cases with surgical intervention for CLE reported in the literature (1–9, 18), only one case of perioperative death has been reported (1–9, 17, 18). In addition, 7/18 reported cases also detail long-term follow-up revealing clinically normal dogs (1–6, 8). Given the reported good short- and long-term outcomes, the literature supports surgical removal of affected lung lobes as treatment of choice for CLE, particularly for clinically affected dogs. Prognosis for the dog of this case report is excellent given the lack of dyspnea or exercise intolerance at 18 months postoperatively, as well as confirmation on preoperative imaging that the remaining lung lobes were within normal limits.

## Data availability statement

The original contributions presented in the study are included in the article/supplementary material, further inquiries can be directed to the corresponding author.

## Ethics statement

Ethical review and approval was not required for the animal study because this is a case report of a clinical case and underwent no changes to clinical care as a result of this case report. Written informed consent was obtained from the owners for the participation of their animals in this study. Written informed consent was obtained from the individual(s) for the publication of any potentially identifiable images or data included in this article.

## Author contributions

CL, SH, and ME were involved in the clinical management of this case. CL, SH, and LE discussed the case. LE was responsible for drafting the manuscript. All authors read and approved the final version of the manuscript.
